# Beneficial Effects of Oat Beta-Glucan Dietary Supplementation in Colitis Depend on Its Molecular Weight

**DOI:** 10.3390/molecules24193591

**Published:** 2019-10-05

**Authors:** Ewa Żyła, Katarzyna Dziendzikowska, Małgorzata Gajewska, Jacek Wilczak, Joanna Harasym, Joanna Gromadzka-Ostrowska

**Affiliations:** 1 Department of Dietetics, Faculty of Human Nutrition and Consumer Sciences, Warsaw University of Life Sciences, Nowoursynowska 159c, 02-776 Warsaw, Poland; ewa_zyla@sggw.pl (E.Ż.); joanna_gromadzka_ostrowska@sggw.pl (J.G.-O.); 2 Department of Physiological Sciences, Faculty of Veterinary Medicine, Warsaw University of Life Sciences, Nowoursynowska 159, 02-776 Warsaw, Poland; malgorzata_gajewska@sggw.pl (M.G.); jacek_wilczak@sggw.pl (J.W.); 3 Adaptive Food Systems Accelerator – Research Centre, Wrocław University of Economics and Business, Komandorska 118/120, 53-345 Wrocław, Poland; joanna.harasym@ue.wroc.pl; 4 Department of Biotechnology and Food Analysis, Wrocław University of Economics and Business, Komandorska 118/120, 53-345 Wrocław, Poland

**Keywords:** oat beta-glucan, colitis, immunomodulation, cytokine, chemokine

## Abstract

Background: Inflammatory bowel diseases are an important health problem. Therefore, the aim of the present study was to compare the impact of isolated oat beta-glucan fractions of low and high molecular weight, taken as dietary supplementation, on inflammatory markers in the colitis model. Methods: Two groups of Sprague–Dawley rats—control and with experimentally induced colitis—were subsequently divided into three subgroups and fed over 21 days feed supplemented with 1% of low (βGl) or high (βGh) molecular weight oat beta-glucan fraction or feed without supplementation. The level of colon inflammatory markers, cytokines, and their receptors’ genes expressions and immune cells numbers were measured by ELISA, RT-PCR, and by flow cytometry methods, respectively. Results: The results showed moderate inflammation affecting the colon mucosa and submucosa, with significant changes in the number of lymphocytes in the colon tissue, elevated cytokines and eicosanoid levels, as well as disruption of the main cytokine and chemokine cell signaling pathways in colitis rats. Beta-glucans supplementation caused a reverse in the percentage of lymphocytes with stronger effects of βGh and reduction of the levels of the inflammatory markers, and improvement of cytokine and chemokine signaling pathways with stronger effects of βGl supplementation. Conclusions: The results indicate the therapeutic effect of dietary oat beta-glucan supplementation in the colitis in evident relation to the molecular weight of polymer.

## 1. Introduction

Proper functioning of the immune system, especially the ability to stimulate cells of this system, is particularly important in immune-mediated inflammatory diseases (IMID) and in neoplastic diseases. Research confirms that patients who have been diagnosed with at least one chronic inflammatory disease are at higher risk of developing other diseases from the IMID group [1,2].

Crohn’s disease (CD) and ulcerative colitis (UC) are two of the most common types of inflammatory bowel disease (IBD) belonging to the IMID. Due to the lack of pathognomonic features and specific diagnostic tests, they are recognized by excluding other disease entities. A common feature of these diseases is their unknown primary cause and complex multifactorial pathogenesis. In the case of CD, a chronic inflammatory process can be located in each section of the digestive tract and cover the entire thickness of the intestinal wall. In the case of UC first of all, the mucosa and submucosa of the large intestine are occupied. 

The clinical features of UC and CD can be distinguished; however, one cannot rule out that a patient diagnosed with a typical phenotype of UC will develop the typical phenotype of CD after several years. The currently available data show that the rate of IBD occurrence is constantly increasing, especially in adolescents [3,4].

At present, it is recognized that CD is mainly a consequence of an immune response to the antigens of the intestinal microflora. Changes in the intestinal bacterial flora, immune factors that cause disorders of the immune system of the gastrointestinal mucosa, and genetic conditions [5] are listed among the factors causing the disease. Moreover, two related mechanisms are involved in the development of the disease. The first one is an oxidative mechanism, which generates an excess of free radicals, and the second is a pro-inflammatory cascade activating the immune response, causing a long-lasting process of inflammation, and resulting in severe lymphatic infiltration of the intestines. Both mechanisms are impaired, and the immune response becomes disrupted. 

The management of CD involves a therapeutic pyramid with several tiers of medication. The basic medications for the treatment of pharmacological CD are preparations of 5-amino-salicylic acid, glucocorticoids, or antibacterial drugs, followed by immunomodulatory and ultimately biological drugs. A new top–down approach to therapy inverts this procedure and presupposes the initiation of the administration of biological or immunomodulatory medications immediately after the diagnosis of the disease to prevent later complications. Preliminary data partially confirm the validity of this concept, but its introduction for everyday practice should be preceded by clinical trials [6]. It seems likely that the development and availability of biological drugs and other new substances will improve the results of treatment of IBD in the coming years for more personalized and targeted treatment.

Beta-glucans are polysaccharides belonging to the soluble fiber fraction, extractable from cereals, mushrooms, seaweed, and yeasts, and classified as physiologically active compounds called biological response modifiers (BRMs). The Food and Drug Administration has included beta-glucans in natural compounds that affect the immune response. The positive impact of beta-glucans on organisms results from their immune-stimulating properties. Beta-glucans have the ability to bind to immune cells receptors, activate them, and regulate the humoral as well as cell-mediated immunity. The anti-inflammatory activity of oat beta-glucan in the upper gastrointestinal tract was shown in our previous studies on the chronic LPS-induced enteritis model [7,8]. 

The immunomodulatory effect of beta-glucans obtained from mushrooms or yeasts in the intestines is quite well documented in scientific literature [9,10]. On the other hand, there are few publications that describe the impact of beta-glucans derived from cereal grains, including oat [11]. Various recent studies have suggested that indigestible polysaccharides represent interesting types of food components that may possess biological activities, and therefore could be beneficial for health. Different origins and methods of extraction result in various lengths of polymer chains; therefore, the diverse scope of metabolic activity of beta-glucans may be not only the result of different chain configurations, but also the distinct molecular weight of the chains of this polymer. A limited number of research studies investigating the health effects of oat beta-glucans with different molecular weight, especially in the context of ongoing inflammation, encouraged us to compare the effects of the pure fraction of oat fiber in the form of low and high molecular weight beta-glucans with or without experimentally induced colon inflammation in Sprague–Dawley rats, as a model that corresponds to the CD that is one of the most widespread types of IBD. A detailed characterization of animal feed and oat beta-glucans was performed to investigate how the molecular weight of beta-glucans affects their biological activity in vivo, which was assessed by animal observations, determination of the level of inflammatory cytokines, and the expression of their specific receptors, as well as investigation of the number of intraepithelial lymphocytes (IELs) and lamina propria lymphocytes (LPLs) in the colon tissue of control and colitis animals that were supplemented with beta-glucans. 

## 2. Results

### 2.1. Feed Consumption and Body Weight Gain

During the first two days after the administration of 2,4,6-trinitrobenzenosulfinic acid (TNBS), the health status of rats from the C group significantly deteriorated, which resulted in decreased food intake, lower activity, and diarrhea. In rats from the H subgroups, no abnormal clinical signs nor behavior changes were noted throughout the experimental period. The rats from subgroup CβG− were in a worse condition, with very common diarrhea, whereas in the animals from groups CβGl+ and CβGh+ fed diet containing beta-glucans, no diarrhea was noted. No exposure-related mortality was registered.

**Figure 1:**
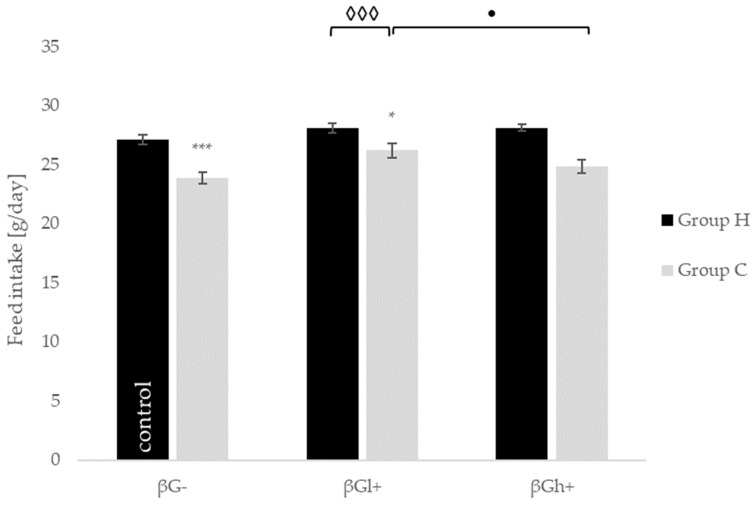
Mean daily feed intake during three weeks of experiments. The data are presented as mean ± SE. Symbol * represents results significantly different from control conditions (HβG−): * *p* < 0.05, *** *p* < 0.001; symbol • represents significant differences between CβGl+ and CβGh+ groups: • *p* < 0.05; symbol ◊◊◊ represents significant differences between healthy (H) and control (C) rats within the same dietary intervention (HβGl+ vs. CβGl+): ◊◊◊ *p* < 0.001.

As shown in Figure 1, daily feed intake during the experimental period was significantly lower in TNBS-treated rats from the CβGl+ (*p* < 0.05) and CβG– (*p* < 0.001) dietary subgroups than in healthy control (HβG−). In the CβGh+ group, such significant differences were not found. These results were confirmed by ANOVA analysis (*p* < 0.001). Moreover, the mean daily feed intake in rats from CβGl+ was significantly higher than that stated in animals from the CβGh+ group (*p* < 0.05).

**Figure 2:**
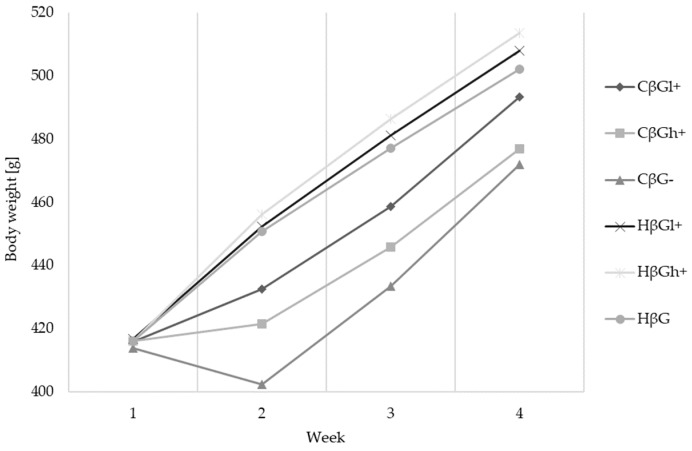
Body weight gain curves of colitis [C] and healthy control [H] groups during three weeks of experiments.

As shown in Figure 2, the initial body weight of rats was similar in all the groups (415.8 ± 11.2 g), and no statistically significant differences in the body weight were observed between the H dietary subgroups during the next three weeks of experiments. Moreover, the body weight of the control rats (H subgroups) systematically increased within three weeks of experiments. The body weight of the TNBS-treated rats (C subgroups) decreased markedly one week post TNBS administration, especially in the group CβG− (*p* < 0.01). During the next two weeks, in all the C dietary subgroups, the body weight increased to a significantly greater extent in the CβGl+ group in comparison to the CβG− group (*p* < 0.05). The final body weight of all the rats was not significantly different, except for significantly lower values for the CβG− and CβGh+ groups (*p* < 0.05).

### 2.2. Macroscopic and Microscopic Changes

**Figure 3:**
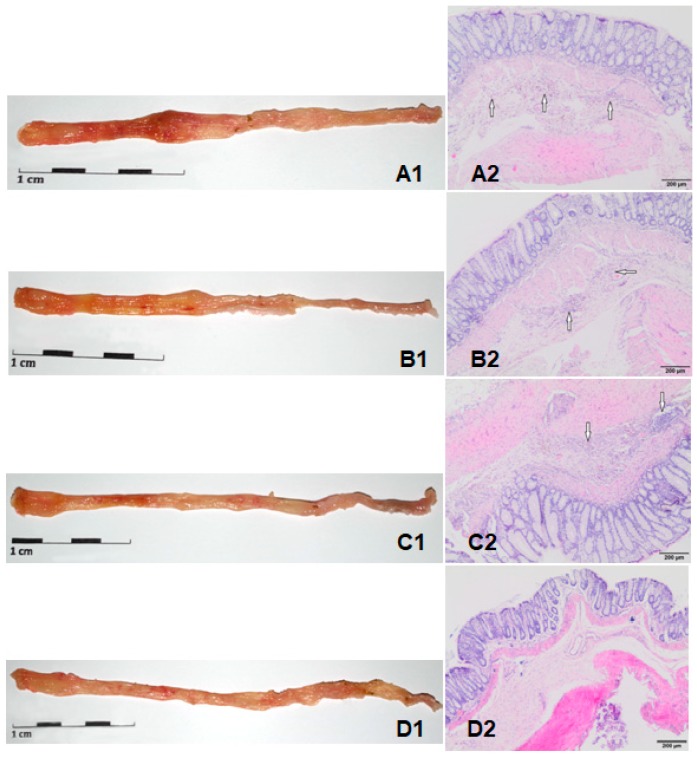
Macroscopic (left side) and microscopic (right side) changes in the colon caused by inflammation. **A1** and **A2**—CβG− group; **B1** and **B2**—CβGh+ group; **C1** and **C2**—CβGl+ group; **D1** and **D2**—HCβG− group. White arrows indicate diffuse multifocal inflammations (lymphocytes infiltration) of the submucosa of varying severity.

The intrarectal injection of TNBS led to local changes in the colon, which were visible during macroscopic evaluation (Figure 3A1) as a score 2 (4 rats), score 3 (2 rats), and score 4 (2 rats). Macroscopic damage included mucosal edema mild or moderate, bleeding ulcers, and erosions. In some cases, tissue necrosis (score 4) was observed. The consumption of feed supplemented with low or high molecular weight oat beta-glucans reduced visible macroscopic lesions (Figure 3B1,C1), which was defined as a score 1 (mucosal erythema only, 7 rats) or score 2 (mild mucosal edema, slight bleeding, or small erosion, 2 rats). The large intestine from control groups (Figure 3D1), regardless of dietary intervention, did not have any pathological changes (score 0, no macroscopic changes). 

**Figure 4:**
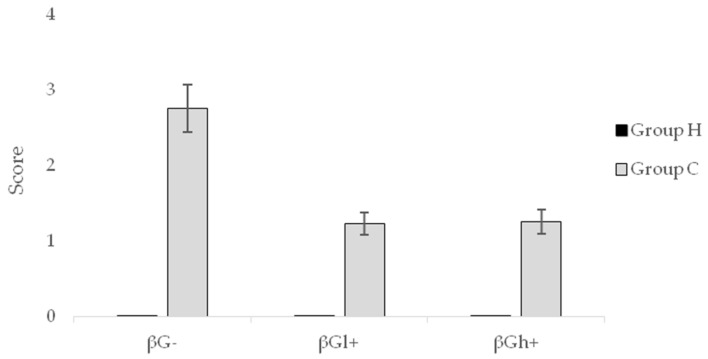
Score of macroscopic damages in the mucosa and submucosa of the colon (mean ± SE).

The mean macroscopic score representing damages in the CβG− group significantly differed from the scores found in the CβGl+ group and the CβGh+ group (*p* < 0.05), (Figure 4, Table 1).

**Figure 5:**
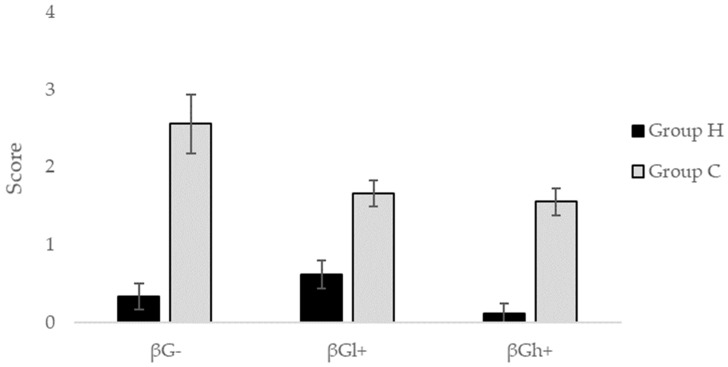
Score of microscopic damages in the mucosa and submucosa of the colon (mean ± SE).

The macroscopic changes presented in Figure 3 were confirmed by microscopic assessment, in which the moderate intensity of multifocal inflammation of the submucosa varied between score 2 (5 rats) or 3 (4 rats) in the CβG− group (Figure 3A2). The histological evaluation of the colons of rats from the CβGl+ and CβGh+ groups revealed a pronounced reduction in the inflammatory response with mild multifocal submucosa inflammation (score 1 in all rats) (Figure 3B2,C2). The normal colonic tissue (score 0) in rats from the HβGl+, HβGh+, and HCβG− groups were observed (Figure 3D2). The mean histopathological score in the CβG− group significantly differed from the scores found in the CβGh+ group (*p* < 0.05), (Figure 5, Table 2).

On the basis of the above macroscopic and microscopic analysis of the colon wall, it should be stated that these damages related mainly to the mucosa or submucosa layers (the CβG− group), and the dietary intervention with beta-glucans significantly decreased the tissue damage with significantly greater damage reduction in the CβGh+ group. 

### 2.3. Influence of Beta-Glucans on Intestinal Lymphocyte Subpopulations

We also investigated the profile of two lymphocyte populations residing in the colon tissue of rats fed differentially supplemented diets of control healthy animals and during ongoing colitis evoked by intrarectal TNBS injection. Flow cytometric analysis enabled us to determine the percentage distribution of CD3+ cells (total T lymphocytes), CD3 + CD8a + cells (Tc lymphocytes), CD+CD4+ cells (Th lymphocytes), CD45RA+ cells (B lymphocytes), and CD161a+ cells (NK cells) within the intraepithelial lymphocyte (IEL) and lamina propria lymphocyte (LPL) populations.

**Figure 6:**
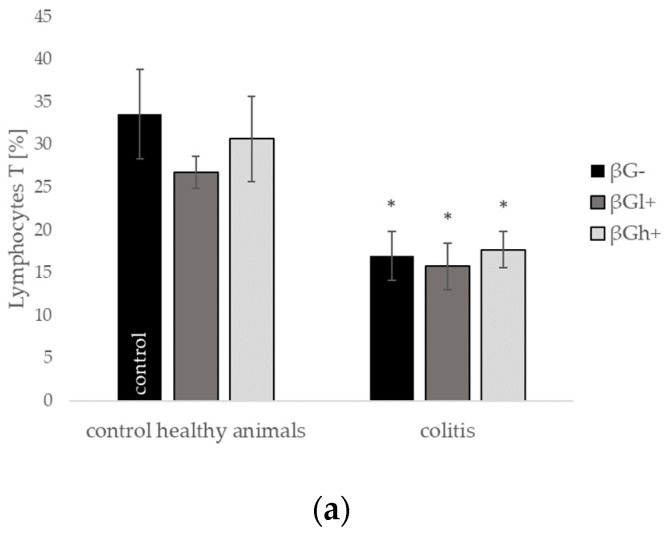

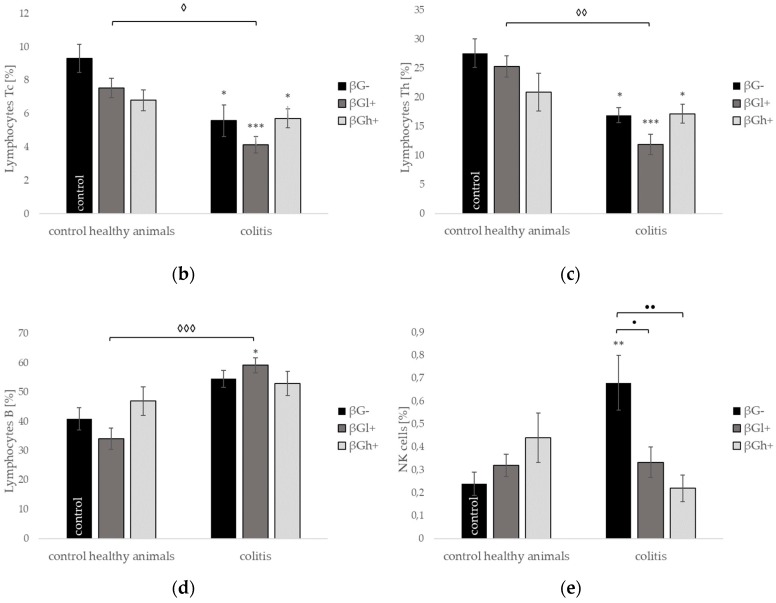
Percentage of (**a**) CD3 cells (lymphocyte T total), (**b**) CD8a+ cells (Tc lymphocytes), (**c**) CD4+ cells (Th lymphocytes), (**d**) CD45RA+ cells (B lymphocytes), and (**e**) CD161a+ cells (NK cells) in the intraepithelial lymphocytes (IEL) population (dithiothreitol, or DTT fraction). Symbol * represents results significantly different from the control group (control healthy animals βG−) (* *p* < 0.05, ** *p* < 0.01, *** *p* < 0.001); symbol • represents significant differences between the colitis βGl+ and colitis βGh+ groups (• *p* < 0.05, •• *p* < 0.01); symbol ◊ represents results significantly different between the control healthy animals and colitis rats within the same dietary intervention (healthy animals βGl+ vs. colitis βGl+) (◊ *p* < 0.05, ◊◊ *p* < 0.01, ◊◊◊ *p* < 0.001); (two-way ANOVA with Tukey post-hoc test); the data are presented as mean ± SE (*n* = 9).

**Figure 7:**
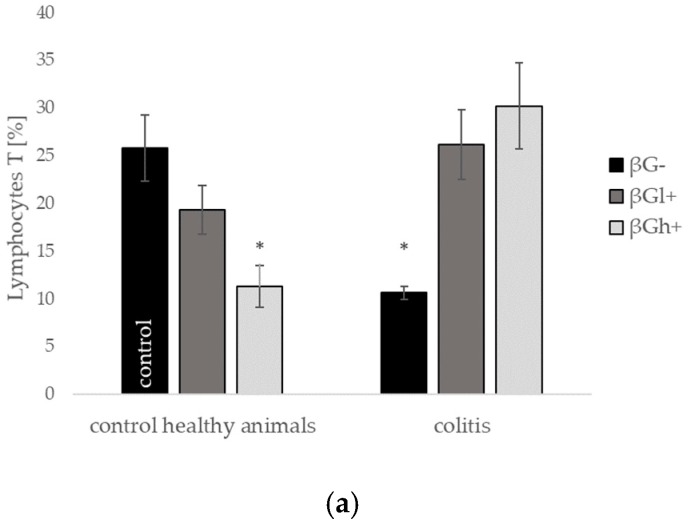

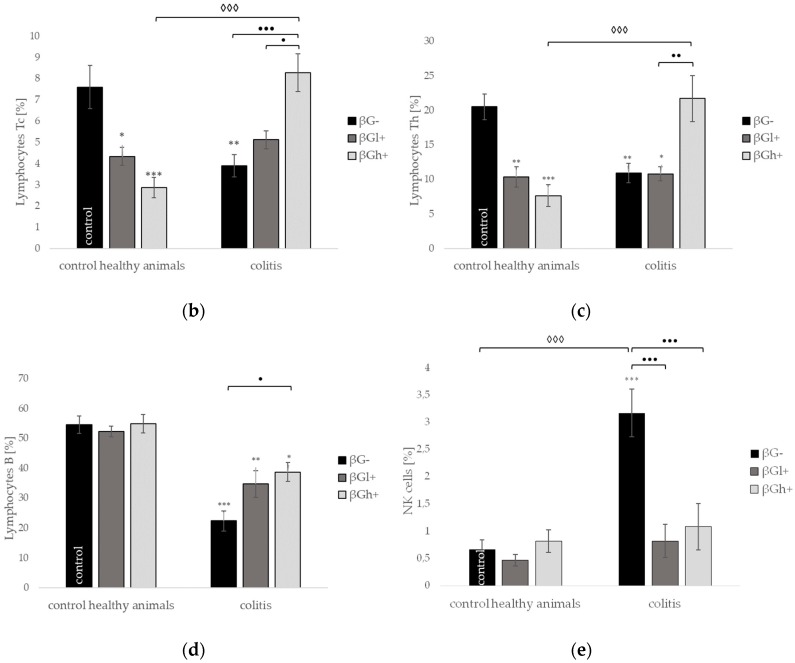
Percentage of (**a**) CD3 cells (lymphocyte T total), (**b**) CD8a+ cells (Tc lymphocytes), (**c**) CD4+ cells (Th lymphocytes), (**d**) CD45RA+ cells (B lymphocytes), and (**e**) CD161a+ cells (NK cells) in the lamina propria lymphocytes (LPL) population (collagenase fraction); symbol * represents results significantly different from the control group (control healthy animals βG−) (* *p* < 0.05, ** *p* < 0.01, *** *p* < 0.001); symbol • represents significant differences between the colitis βGl+ and colitis βGh+ groups (• *p* < 0,05, •• *p* < 0.01, ••• *p* < 0.001); symbol ◊ represents results significantly different between the control healthy animals and colitis rats within the same dietary intervention (healthy animals βGl+ vs. colitis βGl+) (◊◊◊ *p* < 0.001); (two-way ANOVA with Tukey post-hoc test). The data are presented as mean ± SE (*n* = 9).

Rats with ongoing inflammation in the colon tissue from all groups (CβG−; CβGl+; and CβGh+) showed a statistically significant decrease in the percentage of T lymphocytes as well as both T lymphocytes subpopulations: Tc and Th within the IELs population (Figure 6). In the case of T cells detected within the IELs population, feed supplementation with beta-glucans did not significantly change the number of T, Tc, and Th lymphocytes in both healthy animals and in animals with colitis. However, changes in the number of T cells were noted within the LPLs population (Figure 7). LPLs population isolated from the colon tissue of C group rats fed diet supplemented with high molecular weight beta-glucan (the CβGh+ group) showed a markedly increased number of CD4+ and CD8a+ T cells compared with the respective HβGh+ group, whereas administration of the low molecular weight beta-glucan (CβGl+ group) caused an increase only in the number of CD8a+ T lymphocytes (CβGl+ versus HβGl+) (Figure 7). In control healthy animals (HβG− group), feed supplementation with both beta-glucans resulted in a decreased number of Tc and Th lymphocytes cells within the LPLs population (Figure 7). 

The percentage of B lymphocytes increased in the IELs population under the influence of inflammation in animals subjected to supplementation with the low molecular weight oat beta-glucan (CβGl+) group in comparison to the healthy control (HβG−) as well as respective (HβGl+) dietary subgroup (Figure 6). On the other hand, in the LPLs population, the percentage of B cells significantly decreased in all animals with colitis compared to the healthy control (HβG−), and the smallest decrease was observed in the case of supplementation with the high molecular weight beta-glucan (CβGh+).

Rats from groups with TBNS-induced colitis (CβG−) showed also an elevated percentage of NK cells in the colon tissue; the results were significant in the IELs and LPLs population. Beta-glucans supplementation caused a decrease in the number of these cells to the levels observed in control healthy animals (HβG−) (Figure 6 and Figure 7).

### 2.4. Influence of Beta-Glucans on the Chosen Markers of Immunity

**Figure 8:**
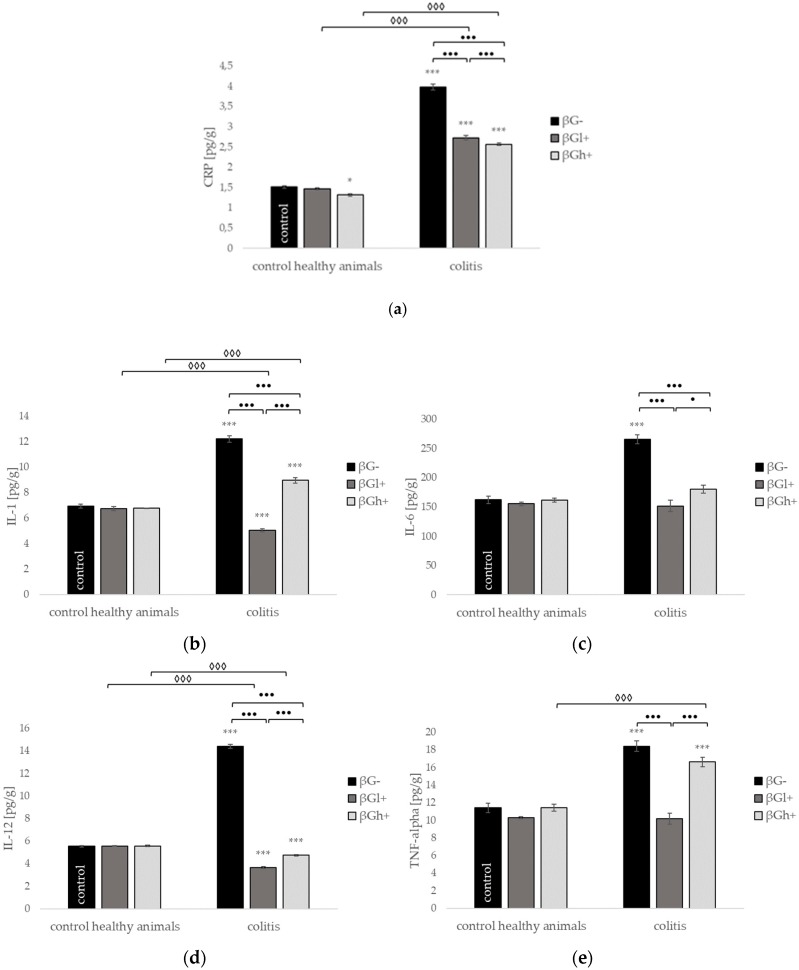
Influence of beta-glucans on the (**a**) C-reactive protein (CRP; (**b**) interleukin 12 (IL-12); (**c**) tumor necrosis factor α (TNF-α); (**d**) interleukin 1 (IL-1); and (**e**) interleukin 6 (IL-6) protein level; symbol * represents results significantly different from the control group (contro healthy animals βG−) (* *p* < 0.05, *** *p* < 0.001); symbol • represents significant differences between the colitis βGl+ and colitis βGh+ groups (• *p* < 0,05, •• *p* < 0.01, ••• *p* < 0.001); symbol ◊ represents results significantly different between healthy and colitis rats within the same dietary intervention (healthy βGl+ vs. colitis βGl+) (◊ *p* < 0.05, ◊◊ *p* < 0.01, ◊◊◊ *p* < 0.001) (two-way ANOVA with Tukey post-hoc test). The data are presented as mean ± SE (*n* = 9).

**Figure 9:**
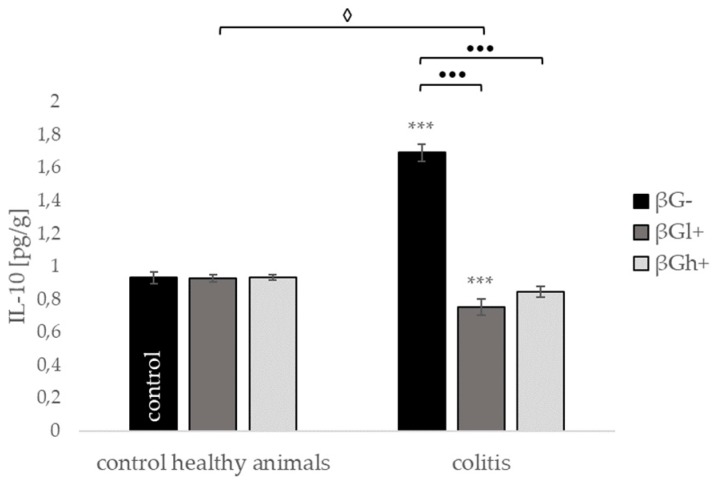
Influence of beta-glucans on the interleukin 10 (IL-10) protein level. Symbol * represents results significantly different from the control group (control healthy animals βG−) (*** *p* < 0.001); symbol • represents significant differences between the colitis βGl+ and colitis βGh+ groups (••• *p* < 0.001); symbol ◊ represents results significantly different between the healthy and colitis rats within the same dietary intervention (healthy animals βGl+ vs. colitis βGl+) (◊ *p* < 0.05) (two-way ANOVA with Tukey post-hoc test). The data are presented as mean ± SE (*n* = 9).

The addition of beta-glucan to the feed of animals in physiological conditions did not change the levels of analyzed cytokines: interleukin 12 (IL-12), tumor necrosis factor α (TNF-α), IL-1, IL-6, and IL-10 (Figure 8 and Figure 9). The C-reactive protein (CRP) protein level significantly decreased in healthy animals with high molecular weight beta-glucan diet supplementation (HβGh+) compared with the healthy control (HβG−). The induction of inflammation by intrarectal TBNS injection without changing the animals’ diet (CβG−) resulted in a statistically significant increase in the levels of all tested inflammatory markers. In the case of CRP, IL-6, IL-12, and IL-10, the addition of both beta-glucans to the feed of animals with induced colitis caused a statistically significant reduction of their level to the level observed in healthy control group (HβG−) or even lower. Additionally, in the case of CRP protein level, we observed a stronger decreasing effect of high molecular weight oat beta-glucan (CβGh+ versus CβGl+; *p* < 0.001), whereas in the case of IL-6 and IL-12, stronger decreases were noted in colitis animals fed diet supplemented with low molecular weight oat beta-glucan (CβGl+ versus CβGh+; *p* < 0.05; *p* < 0.001 respectively). In the case of the TNF-α and IL-1 in colitis groups, the protein level decreased significantly with oat beta-glucan supplementation compared to the colitis group (CβG−), but only the addition of low molecular weight beta-glucan reduced the level of the above cytokines to the value noted in the healthy control group (HβG−).

### 2.5. Selected Inflammatory Markers

**Figure 10:**
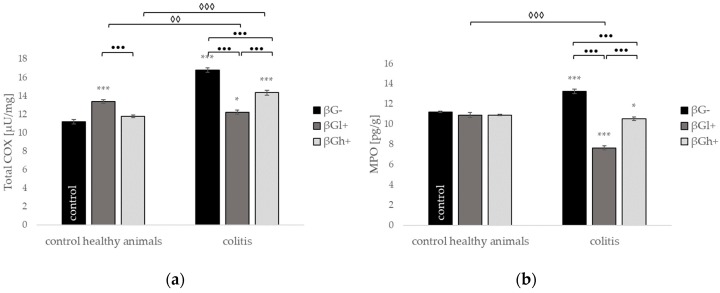

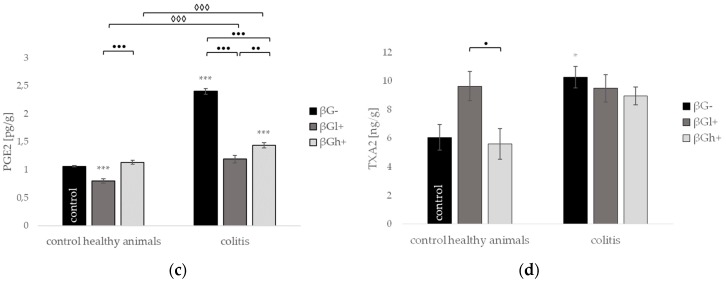
Influence of beta-glucans on the (**a**) total cyclooxygenase (COX) activity; (**b**) tromboksan A2 (TXA2); (**c**) prostaglandin E2 (PGE2); and (**d**) myeloperoxidase (MPO) protein level. Symbol * represents results significantly different from the control group (control healthy animals βG−) (* *p* < 0.05, *** *p* < 0.001); symbol • represents significant differences between the colitis βGl+ and colitis βGh+ groups (•• *p* < 0.01, ••• *p* < 0.001); symbol ◊ represents results significantly different between the healthy and colitis rats within the same dietary intervention (healthy animals βGl+ vs. colitis βGl+) (◊◊ *p* < 0.01, ◊◊◊ *p* < 0.001) (two-way ANOVA with Tukey post-hoc test). The data are presented as mean ± SE (*n* = 9).

Furthermore, we determined the level of proteins associated with the formation of compounds, which are the local cellular information source about phospholipids transformation in the cell membrane. The level of total cyclooxygenase (COX), prostaglandin E2 (PGE2), tromboksan A2 (TXA2), and myeloperoxidase (MPO) in the colon significantly increased in animals with induced colitis without supplementation (CβG− vs. HβG−) (Figure 10). In the case of COX, PGE2 and MPO in groups witch colitis, the level of above compounds decreased significantly in animals fed with diet supplemented with beta-glucans (CβGl+ and CβGh+ versus CβG−), but only the addition of low molecular weight beta-glucan reduced the level of analyzed compounds to the value detected in the healthy control group (HβG−).

### 2.6. Effect of Beta-Glucans on the Expression of Genes Encoding Inflammatory Cytokines and their Receptors

**Figure 11:**
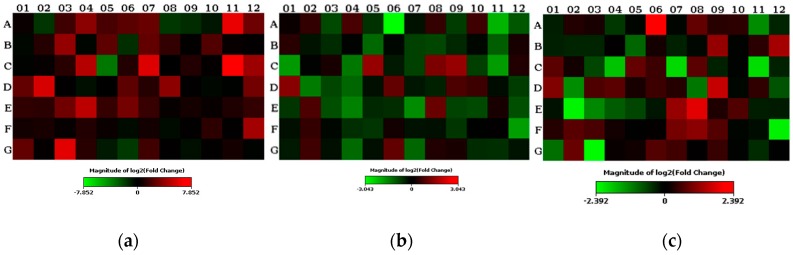
Two-dimensional heat map showing all genes analyzed for each exposure combination, and representing the average gene expression levels. Genes that were up-regulated are shown in red, and genes that were down-regulated are shown in green in the experimental group: (**a**) in the CβG− group vs. HβG−; (**b**) in the CβGl+ group vs CβG−; (**c**) in the CβGh+ group vs CβG−.

To clarify the molecular mechanism behind the observed impact of oat beta-glucan, we analyzed the effects of oat beta-glucan diet supplementation on the expression of 84 different genes that encode inflammatory cytokines and their receptors in the colon tissue. The analysis was done by real-time PCR using the Rat Inflammatory Cytokines and Receptors array, allowing for specific gene profiling (Supplementary Table S1). Results obtained revealed that out of 84 analyzed genes, 22 were up-regulated in samples from the TNBS-induced colitis group (CβG−) when compared with the healthy control group (HβG−). The list of up-regulated genes included those encoding chemokines and their receptors: *Ccl12, Ccl22, Ccr3, Ccr4, Ccr5, Ccr8, Cxcr1, Cxcr2, Cxcr3,* and *Cxcr5*; interleukins and their receptors: *Il13, Il16, Il27, Il2rb, Il2rg, Il6r,* and *Il10ra*; and other inflammatory mediators: *Faslg, Ltb, Osm, Spp1, Tnf,* and *Tnfsf14* (Figure 11a, Supplementary Table S2). The addition of low molecular weight beta-glucan to animal feed (CβGl+ group) significantly up-regulated the expression of *Cxcl1, Il17a, Cxcr1,* and *Spp1* genes, and down-regulated the gene expression of *Ccl19, Cd40lg, Cxcr5, Il10ra, Il16, Il21, Il2rg, Il5ra, Lta, Ltb, Osm, Tnf, Tnfsf11,* and *Tnfsf14* (Figure 11b, Supplementary Table S3). The changes in genes expression were also noted in samples derived from animals fed a diet supplemented with high molecular weight beta-glucan (CβGh+ group). Genes encoding *Cxcl1, Cxcl2, Cxcr2, Il17a, Il1a,* and *Spp1* were up-regulated, whereas *Ccl19, Cd40lg, Cxcr5, Lta, Ltb,* and *Tnfsf11* were down-regulated (Figure 11c, Supplementary Table S3).

## 3. Discussion

In this study, we investigated the effect of oat beta-glucans on experimentally TNBS-induced colitis, which is a well-described intestinal inflammation model with predominantly Crohn’s disease-like features due to the transmural character of the inflammation. Intrarectal administration of TNBS dissolved in 50% ethanol results in the induction of colitis, which mimics human Crohn’s disease (CD) [12,13]. Ethanol disrupts the colon epithelial barrier, enabling the interaction of TNBS with colon’s tissue proteins, which cause transmural inflammation. Both substances together evoke a strong local immune response, which is reflected mostly by lymphocyte T helper 1 (Th1)-mediated inflammation with a dense colonic lymphocytes infiltration and the secretion of pro-inflammatory cytokines. In our study, histological evaluation showed mild or moderate inflammation affecting the mucosa and submucosa layers in rats 21 days after TNBS administration. These features reflect the typical characteristics of Crohn’s disease [14,15]. Moreover, after TNBS administration, the rats from the CβG− group showed significantly lower feed intake and body weight gain, which was connected with full clinical signs, such as bloody diarrhea. We have shown that inflamed rats’ colons responded to 21 days oat beta-glucan dietary intervention in a molecular weight-dependent manner. The greatest inhibition of mucosa and submucosa lymphocytes infiltration occurred after dietary supplementation with high molecular weight oat beta-glucan, while in the case of potential for the recovery of the feed intake and body weight gain, low molecular weight oat beta-glucan was more potent. It can be assumed that high molecular weight beta-glucan, due to its physical properties, forms a protective layer on the internal intestinal wall, which reduces inflammatory damages and decreases the risk of secondary microbial infection. On the other hand, low molecular weight beta-glucan was more effective with reference to the immune cells’ function modulation, as a result of the specific molecular structure of the lower beta-glucan form. The random breaks of the long chain of large molecular weight beta-glucan as a result of nonenzymatic cleavage may change the folding of this polymer. This results in more abundant exposure of the beta-1-3 linkages per mol of substance in beta-glucan with low molecular weight, which is expected to stimulate more intensively the Dectin-1 receptor, and is sensible for linkages of such configuration.

We investigated the immunomodulatory functions of oat beta-glucans using a comprehensive approach in which we analyzed changes in the percentage of immune cells of gut-associated lymphoid tissue (GALT), concentration of pro- and anti-inflammatory cytokines produced within the colon tissue, as well as the expression of genes associated with the immune response. We found that rats with TNBS-induced colitis (CβG−) showed a significant reduction of the number of T lymphocytes in populations of IELs and LPLs in comparison to animals from the healthy control group (HβG−). Intraepithelial lymphocytes are located within the epithelial cells of the GI tract and are essential for controlling the integrity of the epithelium, which is continuously exposed to foreign antigens found in the gut lumen [16]. Lamina propria lymphocytes have been mostly linked with CD4+ Th cells that mediate the effects of the adaptive immune system. More recent studies indicate that LPLs also contains innate lymphocytes, which contribute to regulation of the epithelium integrity and keeping the homeostasis at the time that they maintain the capacity of initiating the proinflammatory responses [16]. Dietary supplementation with low molecular weight beta-glucan (βGl+) did not cause pronounced changes in the percentage of Tc and Th cells in any of the analyzed subpopulations. However, in rats from the colitis group fed diet supplemented with high molecular weight beta-glucan (CβGh+), the percentage of Tc and Th lymphocytes was similar to that observed in animals from the healthy control group (HβG−), and at the same time, it was significantly higher than in the non-supplemented colitis group (CβG-). This may indicate that the oat high molecular weight beta-glucan was capable of stimulating the cell-mediated immune response during the ongoing process of intestinal inflammation. Stimulation of the humoral immune response could also be noted when analyzing the LPLs population, because both beta-glucans significantly increased the percentage of B cells in comparison to the percentage observed in rats from the colitis group not supplemented with these polysaccharides (CβG−). Other research groups investigating the immune effects of beta-glucans derived from different sources also reported an increase in the number of chosen cells of the immune system. Six weeks intervention with orally administered mushroom beta-glucan, lentinan, in healthy human subjects resulted in a significantly elevated number of B lymphocytes in blood [17]. Another study evaluated the effect of active hexose correlated compound (AHCC) on the immune function of healthy adults; it was noted that the frequency of CD4+ and CD8+ T cells in blood increased during AHCC intake, and these values remained increased even 30 days after the experimental period [18]. The difference between the data obtained in our study and the data described above is that we observed a positive effect of oral administration of oat beta-glucans only in groups with induced colitis, but not in the groups not treated with TNBS (healthy). Our results correspond with the data obtained in our previous study, in which we found that the protective effect of beta-glucans on intestinal tissue is observed mainly in the course of ongoing inflammation [8]. This hypothesis is further supported by the results of our present study regarding the percentage of NK cells within the populations of IELs and LPLs. In both populations, the frequency of NK cells was significantly elevated in rats from the non-supplemented colitis group (CβG−), whereas animals from the colitis groups fed diet enriched with beta-glucans (CβGl+ and CβGh+) had the percentage of NK cells similar to animals from healthy control groups which were administered low or high molecular oat beta-glucans (HβGl+ and HβGh+). In addition, we observed a significantly increased level of IL-12 in the tissue of rats with induced colitis (CβG−), which was dramatically reduced in animals from colitis groups subjected to the dietary intervention with beta-glucans (CβGl+ and CβGh+). Since IL-12 belongs to the pro-inflammatory cytokines secreted by NK cells (among others), a connection can be noted between the increased number of NK cells in colitis and the augmented concentration of IL-12 in these conditions. Interestingly, the available literature also presents contradictory data. Rice et al. [19] demonstrated that the oral administration of beta-glucans to mice increased the expression of IL-12. However, their study also confirmed that orally administered beta-glucans improved the survival of *Staphylococcus aureus* or *Candida albicans*-challenged mice, and that orally administered water-soluble glucans translocate from the gastrointestinal (GI) tract into the systemic circulation. This is also in agreement with our results regarding the pathway-focused gene expression analysis, where we found up-regulation of the *Cxcr3* chemokine receptor in rats with colitis that is reported to be associated with the differentiation of Th1 cells and cytokine expression [20].

Immune cells are recruited into the intestinal mucosa during inflammation after their interaction with antigen-presenting cells, such as dendritic cells (DCs). These processes are essential for the differentiation and chemotactic infiltration of pro-inflammatory cells to the site of inflammation. The key mediators in this phenomenon are chemokines produced by DCs [20]. In our present study, 21 days after TNBS exposure (in the CβG− group), we observed the up-regulation of genes encoding chemokines, such as *Ccl12* and *Ccl22*, and chemokine receptors, including: *Ccr3, Ccr4, Ccr5, Ccr6, Cce8, Cxcr1, Cxcr2, Cxcr3,* and *Cxcr5*. Nanki et al. [21] reported that the increased expression of chosen chemokine receptors, such as *Ccr5, Ccr6, Cxcr3,* and *Cxcr5*, is linked with the activity of cytokine-producing B-cells during ongoing inflammation. Our results demonstrated that samples from the experimental groups supplemented with beta-glucans showed the differential expression of chemokine receptors as well as their ligands (*Ccl12-Cxcr2, Ccl22-Ccr4, Cxcl1-Cxcr2,* and *Ccl19-Ccr7*). This may indicate the important role of beta-glucans in modulation of the immune response via chemokines/receptors pathways. Denning et al. [22] also reported that the stimulation of TLR ligands in murine lamina propria macrophages resulted in an increased expression of genes encoding chemokines (*Ccl12, Ccl22*) and their receptors (including *Ccr5*). Furthermore, it has been shown that mice lacking the expression of *Ccl17,* which acts via interaction with the Ccr4 receptor, produced less IL-12 due to blocked chemokine/receptor feedback [23]. 

In our study, changes in the expression of genes encoding immunomodulating cytokines and chemokines were observed simultaneously with the changes in the concentration of analyzed cytokines, which were the highest in the colitis group without beta-glucans supplementation (CβG−). With regard to the pro-inflammatory cytokines IL-1β, IL-6, and TNFα, our results are consistent with the data obtained by others, who showed that the level of pro-inflammatory cytokines increases in animals with colitis induced by exogenous agents [24,25,26]. Increased levels of anti-inflammatory cytokine IL-10 in CβG− may be surprising; however, reports from clinical observations demonstrate a compensatory increase of IL-10 levels in patients with Crohn’s disease [27]. Li et al. [28] also reported a higher concentration of IL-10 in the colon from patients with CD and a concomitant lack of response of mononuclear cells isolated from CD patients to IL-10, which was used as the treatment agent. Interestingly, in our study, the levels of anti-inflammatory cytokine IL-10 and pro-inflammatory IL-1β and IL-6 in the colon tissue of animals treated with TNBS and fed diet supplemented with beta-glucans (CβGl+ and CβGh+) were similar to those observed in healthy rats (HβG−). Additionally, we also observed a strong induction of inflammatory response at the molecular level after TNBS administration. A significant up-regulation of genes encoding the following cytokines and their receptors—*Il13, Il16, Il27, Il2rb, Il2rg, Il6r Il10ra, Faslg, Ltb, Osm, Spp1, Tnf, Tnfsf14*—was noted in animals with colitis (CβG−). All of these signaling molecules are known to be important factors in CD pathophysiology [20,29]. Interestingly, after the addition of beta-glucan to animal feed, we noticed a significant reduction in the expression of *Il10ra, Il16, Il21, Il2rg, Il5ra, Lta, Ltb, Osm, Tnf, Tnfsf11,* and *Tnfsf14* in rats with colitis, with a stronger protective effect demonstrated by low molecular weight beta-glucan (CβGl+). These data may suggest that oat beta-glucans could suppress inflammation by decreasing gene expression and the secretion of proinflammatory cytokines and other inflammatory signaling molecules, and by rebalancing the ratio of pro-/anti-inflammatory cytokines. 

In the case of TNFα, we observed an increased concentration of this pro-inflammatory cytokine also in rats from the colitis group supplemented with high molecular weight beta-glucan (CβGh+). Several reports point out that beta-glucans administration may result in an up-regulated expression of pro-inflammatory cytokines. This effect is most likely derived from macrophages and DCs, which were not analyzed in the present research [30,31]. Elder et al. [30] underline the difference in the effect of small and large particulated beta-glucans in the regulation of cytokines from human DCs. These authors demonstrated that dendritic cells stimulated with large beta-glucan generated significantly more IL-1β, IL-6, and IL-23 compared to those stimulated with the smaller beta-glucans. In our study, the level of IL-1β was also significantly lower in rats from the CβGl+ group in comparison to the level observed in the CβGh+, but the highest concentration was noted in the non-supplemented colitis group (CβG−).

Inflammatory processes are regulated by extracellular mediators, which also include eicosanoids, such as prostaglandin E2 (PGE2). PGE2 is the prostanoid that is most extensively produced upon pro-inflammatory cytokines stimulation, and belongs to the main factors that regulate local inflammatory responses. Although in the physiological state, PGE2 synthesis occurs mainly through the activity of cytosolic and microsomal forms of synthase-2, in the condition of inflammation, PGE2 is produced mainly by microsomal synthase-1 and cyclooxygenase-2 (COX-2) [32]. Boniface et al. [33] revealed that PGE2 is necessary for the production of pro-inflammatory cytokines, and is able to exert two different types of reactions: initiation and the maintenance of inflammation. In our study, we observed an increased level of PGE2 as well as elevated activity of COX in animals with colitis (CβG−). These results correspond with the studies on IBD patients, who showed high activity of COX2 in the epithelium of the large intestine during the active phase of the disease [34]. Interestingly, oat beta-glucans supplementation significantly decreased the concentration of PGE2 in the colon of rats with TNBS-induced colitis. Simultaneously, we observed a close correlation between PGE2 and COX activity, where high cyclooxygenase activity (COX total) translates into a high concentration of PGE2, and points to a key association between PGE2 level and COX activity. The results of our study do not allow us to state unambiguously whether the effect of beta-glucans is based on the inhibition of COX activity (thereby reducing PGE2 synthesis) or through PGE2–beta-glucans interactions, which as a consequence decrease the colon PGE2 concentration. Additionally, we observed that the ability to effectively affect both COX activity and PGE2 level in a higher extent is shown by low molecular weight beta-glucan. Our findings are consistent with the previously reported results of Smeekens et al. [35], who observed a reduction of PGE2 production under the influence of beta-glucan together with decreased mRNA expression and/or protein levels of cytokines such as IL-6, IL-23, IL-10, and IL-17. Smeekens et al. [35] suggest that this effect is mediated by the synergic impact of beta-glucans with dectin-1 and Toll-like receptor 2 (TLR2).

In addition to PGE2 and COX level, we also examined the role of oat beta-glucans on thromboxane A2 (TXA2), which is another strongly pro-inflammatory prostanoid produced due to COX activity. TXA2 increased synthesis is noticed in IBD, and may play an important role in its pathogenesis [36]. In contrast to the concentration of PGE2, TXA2 level was not reduced under the influence of beta-glucans, and remained at a similar level to that observed in the colitis tissues (CβG-). However, the different effects of oat beta-glucans on both COX metabolites suggest that they exert diverse and selective inhibitory properties, which are directed rather toward PGE2 than TXA2. Considering the physiological role of PGE2, this gives grounds to believe that the biological potential of beta-glucans is very wide. Since myeloperoxidase (MPO) is one of the most abundant pro-inflammatory enzymes responsible for the synthesis of hypochlorous acid from hydrogen peroxide and the formation of other highly reactive molecules, such as tyrosyl radicals, and cross-linked proteins, we decided to evaluate MPO activity to expand our understanding of the effects of oat beta-glucans on inflammatory processes. In our study, the activity of MPO was increased under TNBS-treatment (CβG-), and the administration of beta-glucans, especially low molecular weight beta-glucan (CβGl+), decreased MPO activity below the values characteristic for the non-treated animals (HβG−). Similarly, Kim et al. [37] demonstrated that during DSS-induced colitis, MPO activity was increased, and the authors unambiguously confirmed that MPO activity can be used as a substitute and a useful marker of colitis.

Moreover, we found no or very slight effects of beta-glucan in healthy rats. This may suggest that the oat beta-glucan was capable of stimulating or modulating the cell-mediated immune response as well as a humoral immune response only during the ongoing process of intestinal inflammation. Our results indicate that stimulation of the humoral immune response by oat beta-glucan observed in LPLs population and the accompanying decrease in the pro-inflammatory interleukins level occurred only in colitis rats as a result of the inhibiting metabolic activity of the cells. The results of the present study are consistent with our previous observations, which showed that the oat beta-glucan protective effect was less pronounced in animals with healthy colons. The anti-inflammatory activity of oat beta-glucan in the upper gastrointestinal tract was shown in our previous studies also only in animals with LPS-induced enteritis (Suchecka et al., 2014; Wilczak et al., 2015). Similarly, changes in gene expression noticed in the peripheral blood of LPS-treated animals investigated by our group indicate that oat beta-glucans exerted a protective effect in rats with an induced inflammatory state (Błaszczyk et al., 2018). Therefore, we suppose that oat beta-glucans supplementation effectiveness is higher when it is applied in the course of ongoing inflammation as a supporting therapy or treatment factor, rather than as a prophylactic use in a healthy subject.

## 4. Materials and Methods 

### 4.1. Preparation and Characterization of Deproteinated Oat Beta-Glucan Fractions

Beta-glucans of high and low molecular weight were obtained by the methods described in patents [38,39]. In both cases, the alkaline extraction allowed extracting beta-glucan from plant matrices and obtaining a solution from which proteins were removed by precipitation at the isoelectric point. After deproteination, a purified preparation of beta-glucan was obtained, which was subjected to the processes of further removal of residual contaminants. It is crucial to remove the remaining proteins, because their presence may induce metabolic pathways by the presence of e.g., signal peptides. Therefore, obtained preparations were purified using enzymatic treatment by a group of proteolytic, peptidolytic, and amylolytic enzymes. The conditions of the process were appropriately selected to maintain the final fraction of beta-glucans in the range of nominal molecular weight. The molecular mass was determined using HPLC-SEC against commercially available standards of (1-3)(1-4)-beta-d-glucan from oats (Megazyme International). 

### 4.2. Animal Treatment and Experimental Design

An experiment was conducted on 10-week-old adult male Sprague–Dawley rats (*n* = 54) obtained from Charles River Laboratories (Sulzfeld, Germany), with an initial body weight of 400 ± 10 g. After one week of acclimatization, rats were separated into single-housed polycarbonate cages and divided into two main groups: C (*n* = 27) rats with colitis and H (*n* = 27) control healthy animals.

In the animals from group C, distal colon colitis was induced by a single per rectum administration of 1 ml of 2,4,6-trinitrobenzenosulfinic acid (TNBS) (Sigma Aldrich, Darmstadt Germany) solution (150 mg/kg bw) dissolved in 50% ethanol. TNBS administration was done using a polyethylene catheter according to Parra et al. [40]. In the control rats (group H), an equal volume of 0.9% saline solution was administered in the same way. 

After TNBS/saline administration, the rats from both C and H groups were divided into three dietary subgroups (*n* = 9 each) fed pellets feed (ZooLab, Sędziszów, Poland) prepared according to AN-93M formulation [41]. All the semi-synthetic purified feeds were based on natural products (corn starch, maltodextrin, sucrose, α-cellulose, casein, and soybean oil) and a mineral and vitamin mixture recommended by Reeves et al. [41] One feed was supplemented with 1% (w/w) of low molecular mass (1.7 × 10^6^ g/mol, chemical purity 97.4%) oat beta-glucan (feed βGl+; animal groups CβGl+ and HβGl+), second was supplemented with 1% (w/w) of high molecular mass (5.9 × 10^4^ g/mol, chemical purity 99.1%) oat beta-glucan (feed βGh+; animal groups CβGl+ and HβGl+) and the third feed did not have beta-glucan supplementation (feed βG−; animal groups CβG− and HβG−). Feeds composition (proteins, carbohydrates, fat, and fiber) were determined by Mérieux NutriSciences, Silliker, Poland. The determination of (1,3)(1,4)-beta-d-glucan content in feeds was performed according to AOAC 995.16 method (K-BGLU, Megazyme, Ireland) based on lichenase digestion. Due to the presence of previously isolated and highly purified beta-glucan fractions in analyzed samples, the procedure of feed sample dissolution was prolonged to 20 min and supported with intensive mixing using low speed (IKA Turrax, Germany) to facilitate the hydration of the added beta-glucan fraction. The further steps of the procedure, especially enzymatic reactions, were conducted without modifications. The composition of all the experimental and control feeds are shown in Table 3.

All rats were housed in controlled stable environmental conditions (22 ± 1 °C, 50 ± 5% relative humidity, and 12/12 h light/dark cycle, air exchange 15/h) for 3 weeks with feed and water available ad libitum. The feed intake was measured every two days, and body weight was examined once a week. All rats were also observed twice daily for clinical signs.

All experimental procedures were approved by the 2^nd^ Local Animal Care and Use Committee, Warsaw, Poland (Resolution No. 60/2015), according to Polish law regulations and 3R rules (Replacement, Reduction, and Refinement).

### 4.3. Tissue Collection and Preparation

After 21 days of intracolonic infusions and dietary interventions, rats were anesthetized by isoflurane inhalation (Baxter Healthcare, Warsaw, Poland) and bled by cardiac puncture. Just after bleeding, the abdominal cavity was quickly opened, and the segments of distal colons 5 cm in length were removed, opened longitudinally, and rinsed with ice-cold 0.9% saline. The severity of colitis was evaluated by an experienced pathologist according to the criteria of Millar et al. [42] as follows: (0) no macroscopic changes; (1) mucosal erythema only; (2) mild mucosal edema, slight bleeding, or small erosions; (3) moderate edema, bleeding ulcers, or erosions; (4) severe ulceration, erosions, edema, and tissue necrosis.

After macroscopic evaluation, the parts of colons macroscopically damaged were fragmented into similar sizes and collected for analysis. The samples for histological analysis were fixed in 10% buffered formaldehyde, paraffin embedded, cut into 5-µm sections and stained with eosin-hematoxylin for light microscopic evaluation. The colon microscopic damages were evaluated by assigning a score using a scale previously described by Galvez et al. [43], which was as follows: (0) normal colonic tissue; (1) inflammation or focal ulceration limited to the mucosa and submucosa; (2) focal or extensive ulceration or inflammation limited to the mucosa and the submucosa; (3) focal or extensive ulceration and inflammation with involvement of muscularis; (4) focal or extensive ulceration and inflammation with involvement of the serosa; and (5) extensive ulceration and transmural inflammation with involvement of the serosa.

### 4.4. Isolation of Lymphocytes from Colon Tissue

Lymphocytes from colon tissue were isolated according to the method previously described [8]. Briefly, 1–2 cm fragments of colon tissue were collected into centrifuge tubes containing Ca and Mg-free Hank’s balanced salt solution (HBSS) during dissection. In laboratory conditions, tissue samples were flushed with pre-warmed HBSS and incubated in flasks containing 2 mM dithiothreitol (DTT) in Ca and Mg-free HBSS at 37 °C for 30 min. with constant stirring (200 rpm) in order to release intraepithelial lymphocytes (IELs) to the solution. Next, each cell suspension was passed through three layers of sterilized gauze to remove any remaining connective tissues, collected, and kept on ice for further IELs staining. The remaining tissue segments were immersed in 50 mL of 5 mM EDTA in HBSS and shaken vigorously prior the next step of lamina propria lymphocytes (LPLs) isolation. After removing EDTA-HBSS, each segment were incubated in RPMI 1640 medium supplemented with 5% fetal bovine serum, 5.96 g/L of HEPES, antibiotics (100 mg/L of streptomycin sulfate, 7 mg/L of penicillin G potassium), and 2 mg of collagenase type I (125 units/mg) for 1 h at 37 °C with constant stirring (200 rpm) to release leukocytes from the lamina propria in the intestinal mucosa. Then, the suspension was transferred into 15 mL of cold complete RPMI medium. The gauze-filtered suspensions in DTT-HBSS (for IELs) and cell suspensions following the collagenase treatment (for LPLs) were separately centrifuged at 400 g for 5 min at 4 °C. The cells were resuspended in 10 mL of phosphate-buffered saline (PBS) and centrifuged one more time using the same conditions. Finally, the immune cells (two fractions separately) were resuspended in PBS, aliquoted, and prepared for immunofluorescent staining and flow cytometric analyses.

### 4.5. Phenotypic Analysis of the Isolated Leukocytes

Freshly isolated cells from the colon tissue (IELs and LPLs) were stained with antibodies against selected surface markers characteristic for specific lymphocyte subpopulations, using two commercially available sets of antibodies: Rat T Lymphocyte Cocktail (BD PharmingenTM, USA, cat. no: 558493) and Rat T/B/NK Cell Cocktail (BD PharmingenTM, USA, cat no: 558495). After centrifugation (400 g for 5 min at 4 °C), cells were suspended in 100 μL of PBS. Ten microliters of appropriate antibodies mixture were added, and cells were incubated at room temperature for 30 min in dark. After incubation, the cells were washed in 1 mL of PBS, centrifuged at 400 g for 5 min at 4 °C, resuspended in 0.5 mL of PBS, and analyzed using a BD FACSAria™ II flow cytometer (BD Biosciences, USA). The population of lymphocytes was first gated based on morphological characteristics: forward scatter (FSC) and side scatter (SSC) (gate P1). Then, cells located in gate P1 were then analyzed with regard to their positive staining with appropriate antibodies. The results were expressed as a percentage of cells within the gated area (P1). The Th and Tc cell contents were presented as percentage of total CD3+ cells. Data were collected for 20,000 lymphocytes from each sample. Unstained cells were used as negative control. The compensation procedure was made using rat compensation set provided by BD Pharmingen.

### 4.6. Determination of Inflammation Parameters

The samples of colon tissue were homogenized in phosphate-buffered saline just before the analysis. In tissue homogenates, chosen parameters were determined using a competitive specific enzyme immunoassay (ELISA) according to the manufacturer’s instructions (DRG Instruments GmbH, Marburg, Germany). The concentration of pro- and anti-inflammatory cytokines (IL-1, IL-6, IL-10, and IL-12), tumor necrosis factor alpha (TNF-α), C-reactive protein (CRP), prostaglandin E2 (PGE2), thromboxane A2 (TXA2), total cyclooxygenase activity (COX) (Cayman Chemical, Ann Arbor, Michigan, United States), and the activity of myeloperoxidase (MPO) were determined.

### 4.7. RNA Isolation, Reverse Transcription, and Real-Time PCR

Total RNA was extracted from the colon samples using the RNeasy Lipid Tissue Mini Kit (Qiagen, Hilden, Germany) according to the manufacturer’s protocol. RNA concentration and purity was measured using NanoDrop™ 2000 spectrophotometer (Thermo Fisher Scientific, Waltham, MA, USA). Based on the absorbance ratios at A260/A280 and A260/A230, all RNA samples were pure and protein-free. RNA integrity was tested in several randomly selected RNA samples using an Agilent Bioanalyzer 2100 system with the RNA 6000 Nano LabChip® kit (Agilent Technologies, Palo Alto, CA). The samples showed minimum degradation, with an RNA integrity number (RIN) above 9. For PCR analysis, 1 μg of total RNA was converted to complementary DNA (cDNA) in a 20-μL reaction volume using an RT2 First Strand Kit (Qiagen, Hilden, Germany). The cDNA was diluted with 91 μL of UltraPure DNase/RNase-Free distilled water and used for the expression profiling using the RT² Profiler™ PCR Rat Inflammatory Cytokines and Receptors array (Qiagen, Hilden, Germany), according to the manufacturer’s instructions. Briefly, 25 μL of PCR total volume reaction mixture was composed of 1 μL of cDNA template, 11.5 μL of DNase/RNase-Free distilled water, and 12.5 μL of RT2 SYBR Green/ROX qPCR Master Mix with HotStart DNA Taq polymerase, SYBR Green dye, and the ROX reference dye (Qiagen, Hilden, Germany). PCR reaction mixture was used for each well (each primer set) of the PCR array. One technical replicate was made for each sample. PCR amplification was carried out using a Stratagene Mx3005P qPCR (Agilent Technologies, Palo Alto, CA) with an initial 10-min step at 95 °C followed by 40 cycles of 95 °C for 15 s and 60 °C for 1 min. Relative gene expression was calculated using the ΔΔCt method with Ldha and Rplp1 as reference controls. Calculations were done using the Data Analysis Qiagen Center (Qiagen, Hilden, Germany). The results are presented as the relative gene expression of the target gene versus housekeeping gene expression in relation to the healthy control group (HβG−), which was calculated as 1.

### 4.8. Statistical Analysis

Statistical analyses were performed with Statistica software version 13.0 (StatSoft Inc.,Tulsa, OK, USA). Differences between groups were compared using a two-way analysis of variance (ANOVA) followed by a post hoc Tukey’s test. Differences were considered as significant at *p* < 0.05. Results are presented as mean ± SE.

## 5. Conclusions

This study demonstrated the molecular weight-dependent therapeutic effect of dietary oat beta-glucan supplementation in TNBS-induced colitis, which is a suitable Crohn’s disease model. The results revealed stronger soothing inflammation effects of high molecular weight oat beta-glucan fraction supplementation manifested by an inhibition of mucosa and submucosa lymphocytes infiltration due to its ability to form a protective layer on the internal intestinal wall. On the other hand, we have shown the higher potency of a low molecular weight oat beta-glucan fraction in the reduction of the inflammatory markers such as cytokines and eicosanoids protein levels, as well as its ability to modulate the chemokines and cytokines signaling pathways. Our results indicate also that a high molecular fraction of oat beta-glucan is more effective as a specific internal dressing on the inflamed tissue, while a low molecular weight oat beta-glucan fraction modulates the immune cells function on the molecular level. Interestingly, the results of our study revealed that the protective effect of oat beta-glucans was observed only for animals with TNBS-induced colitis, which indicates that the effectiveness of oat beta-glucan supplementation during ongoing inflammation is better preferably than when used as a prevention of inflammatory colon disease.

## Figures and Tables

**Table 1:** Score of macroscopic damages in the mucosa and submucosa of the colon.

Experimental Feeds	H Group	C Group
	Damage score (0–10)	
βG−	0 (0)	2,5 (2–4)
βGl+	0 (0)	1 (1–2)
βGh+	0 (0)	1 (1–2)

Data are expressed as medians (range); *n* = 9.

**Table 2:** Score of microscopic damages in the mucosa and submucosa of the colon.

Experimental Feeds	H Group	C Group
	Damage score (0–6)	
βG−	0 (0–1)	2 (2–5)
βGl+	0 (0–1)	2 (1–2)
βGh+	0 (0–1)	2 (1–2)

Data are expressed as medians (range); *n* = 9.

**Table 3:** Declared and analyzed composition of macronutrients (% w/w) in experimental feeds (for analyzed composition mean ± SD).

Nutrients in Feeds	Declared Composition			Analyzed Composition		
	βGl+	βGh+	βG−	βG+	βGh+	βG−
Protein	14.0	14.0	14.0	12.89 ± 1.80	12.49 ± 1.75	12.75 ± 1.78
Carbohydrates	71.01	71.01	72.01	69.1 ± 2.3	68.1 ± 2.7	69.8 ± 2.7
Fat	4.0	4.0	4.0	3.2 ± 0.1	3.3 ± 0.1	3.5 ± 0.8
Fiber	5.0	5.0	5.0	3.7 ± 0.4	3.6 ± 0.4	3.5 ± 0.3
β-Glucans (%)	1.0	1.0	0.0	0.91 ± 0.076	0.92 ± 0.388	0.0

## Supplementary Materials

The following are available online. Supplementary Table S1. List of investigated genes; Supplementary Table S2. Changes in gene expression in rat colon after 21 days of experiment (compared to control group (HβG-)). Fold regulation higher than 2 and statistically significant in Student’s t-test p-value are highlighted in red (up-regulated genes) or blue (down-regulated genes). Supplementary Table S3. Changes in gene expression in rat colon after 21 days of experiment (comparing to TNBS-colitis induced group (CβG-)). Fold regulation higher than 2 and statistically significant in Student’s *t*-test *p*-values are highlighted in red (up-regulated genes) or blue (down-regulated genes).
