# Supplementary material for: Beneficial Effects of Oat Beta-Glucan Dietary Supplementation in Colitis Depend on Its Molecular Weight
Source: Molecules. 2019 Oct 5;24(19):3591. doi: 10.3390/molecules24193591 (PMC6804032; doi:10.3390/molecules24193591)
Supplement: Supplementary file 1 [file molecules-24-03591-s001.pdf]

Supplementary Table I. List of investigated genes

	1	2	3	4	5	6	7	8	9	10	11	12
A	Aimp1	Bmp2	Ccl11	Ccl12	Ccl17	Ccl19	Ccl2	Ccl20	Ccl22	Ccl24	Ccl3	Ccl4
B	Ccl5	Ccl6	Ccl7	Ccl9	Ccr1	Ccr10	Ccr2	Ccr3	Ccr4	Ccr5	Ccr6	Ccr8
C	Cd40lg	Csf1	Csf2	Csf3	Cx3cl1	Cx3cr1	Cxcl1	Cxcl10	Cxcl11	Cxcl12	Cxcl2	Cxcl6
D	Cxcl9	Cxcr2	Cxcr3	Cxcr5	Faslg	Ifng	Il10ra	Il11	Il13	Il15	Il16	Il17a
E	Il17b	Il17f	Il1a	Il1b	Il1r1	Il1rn	Il21	Il27	Il2rb	Il2rg	Il3	Il33
F	Il4	Il5	Il5ra	Il6r	Il6st	Il7	Cxcr1	Lta	Ltb	Mif	Nampt	Osm
G	Pf4	RGD156 1905 predicted	Spp1	Tnf	Tnfrsf11 b	Tnfsf10	Tnfsf11	Tnfsf13	Tnfsf13b	Tnfsf14	Tnfsf4	Vegfa

Supplementary Table II. Changes in gene expression in rats colon after 21-days of experiment (comparing to control group (HβG-)). Fold Regulation higher than 2 and statistically significant in Student's t-test *p*-value are highlighted in red (up-regulated genes) or blue (down-regulated genes).

**Position**	**Gene Symbol**	**CβGl+**		**CβGh+**		**CβG-**		**HβGl+**		**HβGh+**	
		Fold Regulation	**p-value**	Fold Regulation	**p-value**	Fold Regulation	**p-value**	Fold Regulation	**p-value**	Fold Regulation	**p-value**
A01	Aimp1	1.24	0.186404	-1.00	0.846449	1.17	0.214941	-1.07	0.428100	-1.04	0.743690
A02	Bmp2	-1.71	0.118709	-1.79	0.055770	-1.32	0.197960	1.00	0.906837	-2.10	0.176906
A03	Ccl11	1.03	0.924390	-1.58	0.395095	-1.35	0.188443	-1.47	0.241578	-1.71	0.039777
A04	Ccl12	4.21	0.041559	3.12	0.023312	2.39	0.035153	1.11	0.904422	2.53	0.064302
A05	Ccl17	4.08	0.021083	3.56	0.039228	2.48	0.053750	1.04	0.862759	2.22	0.248913
A06	Ccl19	-3.03	0.469728	-1.33	0.404371	4.69	0.205659	1.84	0.495720	1.08	0.705410
A07	Ccl2	1.51	0.301178	1.45	0.297316	1.28	0.538500	1.04	0.730362	1.19	0.623817
A08	Ccl20	-3.21	0.186954	-2.11	0.521015	-1.01	0.787775	-1.23	0.626524	-1.32	0.868496
A09	Ccl22	2.86	0.088632	4.48	0.082914	4.10	0.002350	1.82	0.113550	3.41	0.002790
A10	Ccl24	1.45	0.208562	1.09	0.713594	1.25	0.410378	1.26	0.413908	1.00	0.901697
A11	Ccl3	1.18	0.952779	3.70	0.026111	2.22	0.151804	1.34	0.478075	1.37	0.443178
A12	Ccl4	1.41	0.460608	2.18	0.116682	2.24	0.089845	1.25	0.436942	1.90	0.124458
B01	Ccl5	1.61	0.307170	1.18	0.758061	1.07	0.910042	-1.60	0.187049	1.39	0.404366
B02	Ccl6	1.08	0.632674	-1.21	0.167655	-1.07	0.499393	-1.27	0.080866	-1.74	0.059605
B03	Ccl7	2.67	0.023493	1.95	0.069749	1.45	0.168158	1.02	0.812800	-1.02	0.830310
B04	Ccl9	-3.79	0.148390	-1.54	0.630800	1.62	0.631122	1.39	0.506775	-1.09	0.562609
B05	Ccr1	1.12	0.933898	1.48	0.160507	1.36	0.317930	1.23	0.537846	1.26	0.521247
B06	Ccr10	1.05	0.471003	1.25	0.428206	1.70	0.049629	1.67	0.042728	1.38	0.298215
B07	Ccr2	2.59	0.001046	2.21	0.001319	1.58	0.007059	1.18	0.248279	1.27	0.190310
B08	Ccr3	2.65	0.000708	2.40	0.001923	2.27	0.019615	1.40	0.159280	-1.11	0.831013
B09	Ccr4	2.90	0.051016	3.15	0.167073	4.32	0.004054	2.41	0.100177	2.44	0.078848

B10	Ccr5	2.31	0.014062	2.09	0.007944	2.03	0.001244	1.31	0.225216	1.48	0.130059
B11	Ccr6	-1.48	0.392506	1.20	0.645148	3.24	0.149329	1.43	0.548835	1.49	0.472437
B12	Ccr8	2.55	0.081220	2.62	0.217259	2.92	0.022668	1.77	0.190080	1.82	0.158388
C01	Cd40lg	-2.65	0.734520	-1.05	0.234447	3.03	0.076185	1.81	0.269396	1.20	0.366420
C02	Csf1	1.61	0.196155	1.59	0.175630	1.65	0.155740	1.73	0.101570	1.36	0.434985
C03	Csf2	1.98	0.041730	1.47	0.093803	1.18	0.222950	-1.17	0.395996	1.28	0.146535
C04	Csf3	1.34	0.311007	2.06	0.132949	1.08	0.786448	-1.08	0.861728	-1.68	0.053195
C05	Cx3cl1	-1.08	0.703884	-1.02	0.649882	1.09	0.780616	2.64	0.188064	1.74	0.357546
C06	Cx3cr1	1.46	0.173069	1.64	0.083247	1.90	0.008004	1.06	0.734798	1.46	0.113812
C07	Cxcl1	3.91	0.033031	4.43	0.056509	1.32	0.398904	-1.02	0.541718	-1.37	0.162276
C08	Cxcl10	2.07	0.037217	1.13	0.592742	1.54	0.119460	1.38	0.258516	2.69	0.060807
C09	Cxcl11	1.71	0.435427	1.15	0.974808	-1.04	0.592500	-1.66	0.189116	2.72	0.162289
C10	Cxcl12	1.23	0.572645	-1.02	0.959852	1.10	0.839841	1.18	0.619576	1.04	0.884936
C11	Cxcl2	2.75	0.202564	14.71	0.172408	1.68	0.371289	1.86	0.187242	1.25	0.893985
C12	Cxcl6	2.81	0.176056	2.81	0.011158	1.62	0.032574	-1.06	0.943924	-4.65	0.533381
D01	Cxcl9	1.22	0.504658	1.40	0.287340	1.75	0.088462	-1.08	0.696412	1.80	0.019157
D02	Cxcr2	3.17	0.016452	7.76	0.082231	2.18	0.092097	1.72	0.180580	2.19	0.026893
D03	Cxcr3	1.40	0.231676	1.60	0.187711	2.01	0.012458	1.55	0.179196	2.14	0.020113
D04	Cxcr5	-2.38	0.444615	1.02	0.567649	3.73	0.045831	2.57	0.115054	1.61	0.348593
D05	Faslg	2.01	0.015447	2.63	0.004122	2.76	0.001691	1.50	0.064434	2.33	0.001802
D06	Ifng	1.39	0.118941	1.27	0.241204	1.37	0.322577	-1.35	0.046611	1.11	0.512072
D07	Il10ra	1.43	0.321661	2.44	0.161893	3.55	0.018503	3.00	0.044051	2.32	0.144963
D08	Il11	1.23	0.250686	1.70	0.162297	1.09	0.593185	-1.20	0.361548	-1.05	0.757455
D09	Il13	4.21	0.010745	4.33	0.083141	3.54	0.016831	2.53	0.261520	2.07	0.197219
D10	Il15	1.19	0.616735	1.06	0.970458	-1.04	0.636887	-1.42	0.118229	-1.02	0.868831
D11	Il16	1.02	0.838718	1.42	0.317840	2.35	0.008879	1.82	0.085309	1.41	0.226926

D12	Il17a	4.50	0.010906	5.51	0.054373	1.57	0.267657	1.83	0.093521	-1.02	0.671475
E01	Il17b	1.08	0.654263	-1.06	0.696054	1.09	0.469745	-1.02	0.999024	-1.35	0.030166
E02	Il17f	2.21	0.059568	1.64	0.074941	1.44	0.130293	1.10	0.898300	-1.07	0.763370
E03	Il1a	2.12	0.608750	3.86	0.081802	1.56	0.840782	1.21	0.834171	2.51	0.330674
E04	Il1b	1.08	0.972163	2.63	0.120451	1.67	0.176491	1.22	0.498148	1.82	0.103701
E05	Il1r1	2.91	0.006592	2.10	0.038543	1.92	0.026736	1.55	0.129183	2.00	0.091170
E06	Il1rn	1.31	0.822806	2.12	0.119818	2.13	0.110863	1.33	0.889324	1.02	0.876593
E07	Il21	1.62	0.169218	2.13	0.173768	4.12	0.072277	1.46	0.223869	1.09	0.905668
E08	Il27	2.18	0.057658	1.89	0.153718	1.99	0.026614	1.36	0.438111	1.23	0.737685
E09	Il2rb	2.01	0.057812	2.22	0.048642	2.80	0.000174	1.64	0.078217	2.20	0.002112
E10	Il2rg	-1.12	0.994175	1.81	0.292299	3.52	0.016672	2.37	0.093913	1.85	0.227675
E11	Il3	1.57	0.088723	1.44	0.124930	1.39	0.155790	-1.07	0.490430	-1.05	0.865105
E12	Il33	-1.07	0.693047	-1.30	0.370567	-1.10	0.608250	1.11	0.571855	-2.17	0.049130
F01	Il4	-1.74	0.202462	-1.01	0.463564	1.76	0.237374	-1.38	0.724167	-1.43	0.411264
F02	Il5	2.98	0.004264	2.07	0.089351	1.58	0.152919	1.07	0.564846	-1.14	0.662198
F03	Il5ra	-1.18	0.380658	1.57	0.263892	2.54	0.062425	2.38	0.089272	1.37	0.416792
F04	Il6r	1.32	0.312716	1.91	0.120517	2.36	0.005620	2.08	0.028838	1.66	0.123975
F05	Il6st	1.91	0.026665	1.69	0.010026	1.69	0.003679	1.37	0.136302	1.30	0.204306
F06	Il7	1.19	0.302979	1.09	0.555028	1.12	0.401632	-1.12	0.467483	1.22	0.297728
F07	Cxcr1	5.44	0.004152	2.12	0.127925	2.44	0.020985	-1.02	0.993720	1.67	0.142870
F08	Lta	-2.15	0.352408	-1.39	0.724287	3.32	0.099230	1.75	0.191314	1.56	0.525708
F09	Ltb	-1.43	0.604151	1.24	0.422368	3.25	0.025209	1.92	0.163948	1.24	0.406922
F10	Mif	1.57	0.001580	1.48	0.006498	1.33	0.013804	1.05	0.721106	1.24	0.161111
F11	Nampt	1.05	0.859316	-1.25	0.129365	-1.09	0.387699	1.09	0.616044	-1.12	0.444501
F12	Osm	-1.08	0.791748	5.52	0.222872	3.09	0.002875	2.29	0.049555	1.59	0.199564
G01	Pf4	1.14	0.438423	-1.14	0.823414	-1.08	0.956780	-1.43	0.146763	-1.52	0.039122

G02	RGD1561905_predicted	-1.69	0.267811	-1.88	0.133473	-1.96	0.096622	1.16	0.358967	-2.16	0.091151
G03	Spp1	7.55	0.086514	14.47	0.114119	2.28	0.032984	-1.07	0.702431	1.98	0.091221
G04	Tnf	1.28	0.580395	2.36	0.173381	2.83	0.036339	2.17	0.131467	1.93	0.277867
G05	Tnfrsf11b	-1.35	0.171615	-1.69	0.111053	-1.37	0.096654	-1.01	0.773119	-1.42	0.190188
G06	Tnfsf10	1.65	0.076008	1.26	0.315122	1.28	0.280030	1.28	0.287855	1.62	0.136366
G07	Tnfsf11	-3.78	0.182867	-1.57	0.904464	2.05	0.462279	1.47	0.509443	1.22	0.687693
G08	Tnfsf13	1.30	0.024084	1.15	0.271207	1.15	0.201282	1.04	0.729425	-1.22	0.307097
G09	Tnfsf13b	1.21	0.256790	1.24	0.154303	1.42	0.003254	1.12	0.387317	1.18	0.135255
G10	Tnfsf14	1.60	0.336967	2.12	0.176895	3.34	0.005982	2.60	0.060138	2.17	0.062094
G11	Tnfsf4	1.59	0.228004	1.21	0.624387	1.58	0.179910	1.03	0.713532	1.16	0.901454
G12	Vegfa	-1.07	0.909128	-1.15	0.886766	-1.08	0.812251	1.11	0.691151	-1.16	0.576638

Supplementary Table III. Changes in gene expression in rats colon after 21-days of experiment (comparing to TNBS-colitis induced group (CβG-)). Fold Regulation higher than 2 and statistically significant in Student's t-test *p*-value are highlighted in red (up-regulated genes) or blue (down-regulated genes).

Position	Gene Symbol	CβGl+		CβGh+	
		Fold Regulation	**p-value**	Fold Regulation	**p-value**
A01	Aimp1	1.06	0.740850	-1.17	0.391242
A02	Bmp2	-1.30	0.535977	-1.36	0.376709
A03	Ccl11	1.39	0.177341	-1.17	0.871778
A04	Ccl12	1.76	0.117710	1.31	0.264688
A05	Ccl17	1.65	0.334025	1.44	0.367066
A06	Ccl19	-14.22	0.223340	-6.24	0.618256
A07	Ccl2	1.18	0.721449	1.14	0.558107
A08	Ccl20	-3.16	0.150466	-2.08	0.641272
A09	Ccl22	-1.43	0.768409	1.09	0.530900
A10	Ccl24	1.16	0.696311	-1.15	0.632146
A11	Ccl3	-1.88	0.178999	1.67	0.127991
A12	Ccl4	-1.59	0.272300	-1.03	0.739761
B01	Ccl5	1.50	0.222998	1.10	0.548467
B02	Ccl6	1.16	0.305008	-1.12	0.255575
B03	Ccl7	1.84	0.064356	1.34	0.261225
B04	Ccl9	-6.14	0.019536	-2.50	0.291978
B05	Ccr1	-1.22	0.255324	1.09	0.374408
B06	Ccr10	-1.62	0.468996	-1.36	0.282362
B07	Ccr2	1.64	0.018821	1.40	0.052748
B08	Ccr3	1.17	0.696514	1.06	0.912485
B09	Ccr4	-1.49	0.283123	-1.37	0.851653

B10	Ccr5	1.14	0.439528	1.03	0.687145
B11	Ccr6	-4.81	0.074588	-2.70	0.337902
B12	Ccr8	-1.14	0.762314	-1.11	0.668351
C01	Cd40lg	-8.01	0.123867	-3.18	0.931046
C02	Csf1	-1.02	0.828170	-1.04	0.976277
C03	Csf2	1.68	0.090314	1.24	0.298239
C04	Csf3	1.25	0.356120	1.91	0.143683
C05	Cx3cl1	-1.18	0.893053	-1.11	0.835683
C06	Cx3cr1	-1.30	0.258445	-1.16	0.641834
C07	Cxcl1	2.96	0.092824	3.35	0.092887
C08	Cxcl10	1.35	0.363263	-1.36	0.255854
C09	Cxcl11	1.77	0.123300	1.19	0.451247
C10	Cxcl12	1.12	0.670149	-1.11	0.805613
C11	Cxcl2	1.64	0.691038	8.75	0.187978
C12	Cxcl6	1.74	0.275925	1.74	0.082934
D01	Cxcl9	-1.43	0.597635	-1.25	0.477467
D02	Cxcr2	1.46	0.436695	3.57	0.115642
D03	Cxcr3	-1.43	0.611421	-1.25	0.590020
D04	Cxcr5	-8.88	0.033396	-3.65	0.381845
D05	Faslg	-1.37	0.279653	-1.05	0.837438
D06	Ifng	1.01	0.748147	-1.08	0.587615
D07	Il10ra	-2.49	0.222526	-1.45	0.521189
D08	Il11	1.13	0.425045	1.56	0.189898
D09	Il13	1.19	0.632808	1.22	0.355023
D10	Il15	1.24	0.023367	1.10	0.104386
D11	Il16	-2.31	0.051771	-1.66	0.509454

D12	Il17a	2.87	0.089601	3.52	0.114280
E01	Il17b	-1.02	0.755154	-1.16	0.331261
E02	Il17f	1.53	0.138655	1.13	0.418447
E03	Il1a	1.36	0.695152	2.48	0.087604
E04	Il1b	-1.54	0.247687	1.58	0.256200
E05	Il1r1	1.51	0.125730	1.09	0.472645
E06	Il1rn	-1.63	0.148316	-1.01	0.635822
E07	Il21	-2.54	0.185901	-1.94	0.468437
E08	Il27	1.09	0.515695	-1.06	0.862638
E09	Il2rb	-1.39	0.465141	-1.26	0.614262
E10	Il2rg	-3.93	0.052633	-1.95	0.514999
E11	Il3	1.13	0.272016	1.03	0.718182
E12	Il33	1.03	0.938560	-1.18	0.598286
F01	Il4	-3.08	0.180876	-1.78	0.674498
F02	Il5	1.89	0.039275	1.31	0.299342
F03	Il5ra	-2.99	0.069435	-1.62	0.230183
F04	Il6r	-1.79	0.269169	-1.24	0.612609
F05	Il6st	1.13	0.355965	-1.00	0.903558
F06	Il7	1.06	0.534381	-1.03	0.791216
F07	Cxcr1	2.23	0.053990	-1.15	0.720578
F08	Lta	-7.15	0.041973	-4.61	0.321846
F09	Ltb	-4.65	0.035946	-2.61	0.296937
F10	Mif	1.18	0.043769	1.11	0.242965
F11	Nampt	1.14	0.181517	-1.14	0.225455
F12	Osm	-3.35	0.032674	1.79	0.303651
G01	Pf4	1.24	0.451304	-1.06	0.882767

G02	RGD1561905_predicted	1.16	0.551272	1.04	0.783228
G03	Spp1	3.31	0.130653	6.35	0.124791
G04	Tnf	-2.21	0.261655	-1.20	0.916888
G05	Tnfrsf11b	1.01	0.965383	-1.23	0.699850
G06	Tnfsf10	1.29	0.374466	-1.02	0.996695
G07	Tnfsf11	-7.76	0.050934	-3.23	0.445767
G08	Tnfsf13	1.13	0.195955	-1.00	0.934611
G09	Tnfsf13b	-1.18	0.709282	-1.15	0.313742
G10	Tnfsf14	-2.08	0.066818	-1.57	0.328659
G11	Tnfsf4	1.00	0.956135	-1.31	0.312138
G12	Vegfa	-1.07	0.909128	-1.15	0.886766
